# Relationship Between Valence and Arousal for Subjective Experience in a Real-life Setting for Supportive Housing Residents: Results From an Ecological Momentary Assessment Study

**DOI:** 10.2196/34989

**Published:** 2023-01-25

**Authors:** Rajesh Nandy, Karabi Nandy, Scott T Walters

**Affiliations:** 1 Department of Biostatistics and Epidemiology School of Public Health University of North Texas Health Science Center Fort Worth, TX United States; 2 Peter O’Donnell Jr. School of Public Health University of Texas Southwestern Dallas, TX United States; 3 School of Public Health University of North Texas Health Science Center Fort Worth, TX United States

**Keywords:** permanent supportive housing, circumplex model of affect, ecological momentary assessment, emotion, valence, arousal, mobile phone

## Abstract

**Background:**

The circumplex model of affect posits that valence and arousal are the principal dimensions of affect. The center of the 2D space represents a neutral state of valence and a medium state of arousal. The role of valence and arousal in human emotion has been studied extensively. However, no consistent relationship between valence and arousal has been established. Most of the prior studies investigating the relationship have been conducted in relatively controlled laboratory settings.

**Objective:**

Ecological momentary assessment (EMA) of affect from participants residing in permanent supportive housing was used to study the relationship between valence and arousal in real-life settings. The goal of this study was to explore the relationship between valence and arousal in a person’s natural environment.

**Methods:**

Participants were recruited from housing agencies in Fort Worth, Texas, United States. All participants had a history of chronic homelessness and reported at least one mental health condition. A subset of participants completed daily (morning) EMAs of emotions and other behaviors. The sample comprised 78 women and 77 men, and the average age was 52 (SD 8) years. From the circumplex model of affect, the EMA included 9 questions related to the participant’s current emotional state (*happy*, *frustrated*, *sad*, *worried*, *restless*, *excited*, *calm*, *bored*, and *sluggish*). The responses were used to calculate 2 composite scores for *valence* and *arousal*.

**Results:**

Statistical models uniformly showed a dominant linear relation between valence and arousal and a significant difference in the slopes among races. None of the other effects were statistically significant. Compared with previous studies, the effects were quite robust.

**Conclusions:**

Our findings may provide a window to the fundamental structure of affect. We found a strong positive linear relationship between valence and arousal at the nomothetic level, which may provide insight into a universal structure of affect. However, the study needs to be replicated for different populations to determine whether our findings can be generalized beyond the population studied here.

## Introduction

### Background

Affect is a fundamental notion in psychology that refers to the occurrence of emotion in the human mind. In fact, affect converges with the conventional definition of cognition such that the differences between affect and cognition are more philosophical than ontological, especially from a neurobiological standpoint [1]. Affect is believed to exist in a 2D space: a dimension for valence and another for arousal [2-6]. Valence is typically defined as an individual’s subjective experience on a positive-negative scale [7]. Arousal is the psychological state of being active as determined by the initiation of the nervous system or, more frequently, by self-report [8]. Sometimes, the dimensional model of affect includes a smaller, third dimension called *dominance* [9,10]. However, valence and arousal are the 2 larger, more persistent dimensions, and much of the literature uses only these 2 dimensions. In this paper, we explore the relationship between valence and arousal from the subjective experience of a group of underserved individuals classified as high risk in a 2D model of affect.

A circumplex model of affect was developed based on the assumption that valence and arousal are the principal dimensions of affect [5]. This model posits that emotions can be represented as points in a 2D space, with arousal captured on the vertical axis and valence captured on the horizontal axis. The center of the 2D space represents a neutral state of valence and a medium state of arousal [11]. The points are scaled such that they are all confined within a circle in the 2D space. Several refinements of the model have been made since its inception [12-15]. A formal quantitative formulation of the circumplex model was introduced recently [16]. It should be noted that the placement of a specific emotion within the circumplex is universal and does not depend on specific population characteristics.

The role of valence and arousal in human emotion has been studied extensively [6]; for instance, Pribram and McGuinness [8] investigated arousal, activation, and effort in the control of attention. Using functional magnetic resonance imaging and behavioral studies, Kensinger and Corkin [17] found that different cognitive and neural processes were associated with enhancing emotional memory to arouse facts compared with valenced nonarousing facts. Experimental evidence also supports the existence of unique neural systems that correspond to the 2 dimensions of affect [18]. The research attempting to connect cognitive scope with affect has generally found that negative affective states limit cognitive range, whereas positive affective states enhance cognitive range [19-21]. Although this consensus generally holds for affects of moderate intensity, there is evidence that affects of low intensity tend to enhance cognitive scope, whereas those of high intensity limit cognitive scope, irrespective of whether the affect is positive or negative [3]. Jhean-Larose et al [22] investigated how these 2 factors lay out activation within the semantic network. Jefferies et al [23] studied the influence of changes in both valence and arousal on the emotion-attention relationship. Mackenzie et al [24] investigated associations among affect, mindfulness, and patient-reported mental health outcomes in a 7-week yoga program for cancer survivors. Mirandola and Toffalini [25] studied how valence and arousal affect false memory production, although positive affect is generally believed to restore self-control resources after depletion [26]. Nealis et al [27] further examined the specific roles of valence and arousal in this effect. Finally, in another recent work, Nandy et al [28] identified behaviors that predicted valence and arousal in the morning for people living in permanent supportive housing (PSH) using ecological momentary assessment (EMA).

Although valence and arousal are represented as individual concepts in the circumplex model, the research support for this assumption is limited. Kuppens et al [29] argued that the nature of the association between valence and arousal is important for 4 reasons. First, a strong association between valence and arousal would suggest that the 2 cannot be influenced independently. This would have profound implications for many psychology experiments that attempt to manipulate and measure emotion. Second, the implication of this finding on the biology of the brain would also be substantial, particularly with respect to attempts to localize valence to brain regions such as the orbitofrontal cortex and arousal to brain regions such as the amygdala [30]. Such attempts rely on the assumption that valence and arousal operate independently of each other. Third, if there is a relationship between valence and arousal, it is critical to ascertain whether it is stimulus dependent; for instance, a V-shaped function of arousal in relation to valence has been observed in affective ratings of visual scenes [31], but it is unknown whether this relationship holds in broader contexts (eg, in the absence of controlled stimulus). Finally, according to Kuppens et al [29], it is important to study the consistency of the relationship across participants to explain variability in the human emotional experience as well as how emotional affect relates to various aspects in the psychological domain. To summarize, it is critical to examine the relationship between valence and arousal from both idiographic (individual participant level) and nomothetic (group level) perspectives.

In establishing a relationship between arousal and valence, Kuppens et al [29] considered seven potential forms drawn from existing psychological theories: (1) independent of each other [32,33]; (2) a linear relationship [34]; (3) a V-shaped relationship [35]; (4-6) an asymmetric V-shaped relationship [36] that includes positivity offset, negativity bias, or both; and (7) a nonparametric regression model. To obtain a comprehensive answer to the question of whether there is a relationship between valence and arousal, the authors analyzed eight data sets: (1) aggregated affective experiences in response to International Affective Picture System (IAPS) pictures [37], (2) data from college students in the United States and Canada that assessed reactions to response items and schemes developed by Barrett and Russell [38], (3) data from college students (the same data set mentioned in the previous point) that assessed feelings resulting from recalls of the past (the last time the participant experienced a strong emotion), (4) data from participants who were given slides from the IAPS picture set and were evaluated on valence and arousal using the Self-Assessment Manikin rating scale [39], (5) similar data from participants using balanced IAPS pictures with responses measured using a Likert scale [40], (6) data from participants reacting to modern art paintings covering a broad affective spectrum, (7) experience sampling from a group of European participants [41] where the participants marked their responses on an affect grid [42], and (8) experience sampling data from American participants [40]. Importantly, only 1 data set (aggregated affective experiences in response to IAPS pictures) was suitable for studying a nomothetic relationship because each participant responded only once, but the other data sets were suitable for studying both nomothetic and idiographic relationships because the participants responded multiple times.

All 7 aforementioned potential forms of the relationship between valence and arousal were considered by Kuppens et al [29] for each data set, and the Bayesian information criterion was used to determine the best-fit relationship [43]. At the nomothetic level, the authors found that the asymmetric V-shaped relationship was the best choice for all data sets (except in the case of data from participants who were given slides from the IAPS picture set and were evaluated on valence and arousal using the Self-Assessment Manikin rating scale, where a positive linear relation was preferred). The consistency of the findings across studies is noteworthy, although the authors acknowledge the weak predictive power of the models with very low *R*^2^ values for the models.

The weak relationship at the nomothetic level was explained by the authors as an artifact of large individual differences at the idiographic level. At the idiographic level, the authors observed that the relationship between valence and arousal can take any arbitrary form or none at all. Because of the large observed differences at the idiographic level, Kuppens et al [44] studied the effects of personality and culture in a follow-up study. From this, the authors concluded that valence and arousal do not have invariable roles in other psychological areas. More fundamentally, the authors did not find evidence for a global model that explains the relationship between valence and arousal in subjective experiences. In fact, the authors recommended that researchers refrain from putting too much faith in the role of affect toward “emotion, well-being, personality, attitudes, moral judgment, memory, perception, and so on.”

As acknowledged by Kuppens et al [44], the first 6 data sets were too limited to draw conclusions about a universal architecture connecting valence and arousal. Notably, each of these data sets involved presentation of visual scenes in a controlled laboratory environment. Although a controlled scientific experiment has its obvious benefits, in this case it is unclear whether the observed relation is intrinsic to the nature of affect or whether it is specific to visual perceptions. Only the last 2 data sets involve affective experiences in a person’s natural environment. In addition, data gathered in an artificial context have limited ecological validity [45]. If the purpose is to draw conclusions about a universal architecture connecting valence and arousal, it is preferable to have responses gathered in familiar, real-life settings.

### Daily EMA Data on Affect

We address this by investigating the relationship between valence and arousal using EMA data. EMA involves repeated sampling of participants’ current behaviors and experiences in real time in the participants’ natural environments [28,45,46]. EMA uses mobile devices to evaluate thoughts, feelings, and behaviors in real time in people’s usual, everyday surroundings. Some form of EMA has been used previously to study the relationship between valence and arousal [40,41]. In this paper, we use daily EMA data on affect from participants residing in PSH who were participating in a health coaching program. People residing in PSH encounter many challenges in being able to live independently, including having various physical and mental health conditions.

## Methods

### Design and Study Procedures

Data for this study were drawn from the Mobile Community Health Assistance for Tenants project, a health coaching intervention designed to improve health indicators among PSH residents in Fort Worth, Texas, United States [47]. Participants were recruited via convenience sampling from 6 local housing agencies in Fort Worth. Participants were adults, English speaking, Medicaid enrolled or eligible, and reported at least one of the following conditions in the past year: prescribed medication for psychological or emotional problems, experienced hallucinations, received a pension for a psychiatric disability, or reported at least moderate levels of depression (a score of >9 on the Patient Health Questionnaire-9).

Participants met monthly with a health coach who helped to set goals related to diet, exercise, substance use, medication compliance, social support, and recreation or leisure. A subgroup of participants who scored ≥4 on the Rapid Estimate of Adult Literacy in Medicine–Short Form test (indicating >grade 6 English literacy level) were given the opportunity to participate in the EMA portion of the project. Each morning, this subset of participants completed EMAs that included questions about current emotions and setting as well as health behaviors from the previous day, including diet, exercise, substance use, leisure time activities, medication compliance, and social interactions.

The EMA protocol used in this study was based on that of a previous study with smokers experiencing homelessness and used in our prior study with the same data set [48]. Participants were provided with a smartphone and granted unlimited voice and text plans as well as 2 GB data for their personal use. For completing the EMAs, participants could earn up to US $15 per month in vouchers redeemable for health-related supplies, proportional to the percentage of days they completed the assessments. Provided that they were compliant with at least 50% of the EMA prompts, participants could retain the smartphone for up to 12 months. The project resources allowed for up to 80 participants to participate in the EMA portion at any one time; when participants returned the smartphone (because they had reached the end of their allotted time, were failing to complete assessments, or had decided that they did not want to carry it any longer), we performed a factory reset on the device and offered it to another participant based on the order of enrollment into the parent study.

### Ethics Approval

The project was approved by the institutional review board of the University of North Texas Health Science Center (approval number 2014-125), and participants were given assurances of confidentiality. All participants provided informed consent.

### Sample

This analysis includes 155 participants who completed 18,357 daily assessments between May 1, 2016, and April 30, 2017. On average, the individuals received 139 (SD 93) daily assessments or prompts and completed 118 (SD 92) assessments. The sample was split almost evenly between men (77/155, 49.7%) and women (78/155, 50.3%), and the average age was 52 (SD 8) years. The racial composition of the 155 participants was as follows: 74 (47.7%) White, 72 (46.4%) African American, 3 (1.9%) American Indian, and 6 (3.9%) in other racial categories. Of the 155 participants, 9 (5.8%) were of Hispanic ethnicity. For statistical analysis, all participants of color were pooled in a single category.

### Instruments (Measures)

#### Overview

The EMA protocol was executed using a smartphone app provided to the participants. The app alerted participants to complete an assessment 30 minutes after their self-reported waking time. Participants were asked to complete the assessment within 30 minutes of the initial alert; they had the option to *snooze* an assessment request 3 times each day before the EMA would be counted as missed. In the next section, we list the questions that were presented in the daily EMA (only the questions or response options considered in our analyses are presented).

#### Emotions

Nine items related to each participant’s current affect (“I feel...”) were measured on a Likert-type scale from 1 (strongly disagree) to 5 (strongly agree): I feel *happy*, I feel *frustrated*, I feel *sad*, I feel *worried*, I feel *restless*, I feel *excited*, I feel *calm*, I feel *bored*, and I feel *sluggish*. Similar EMA items and procedures have been used in previous studies [28,49].

### Statistical Modeling and Analysis

**Figure 1:**
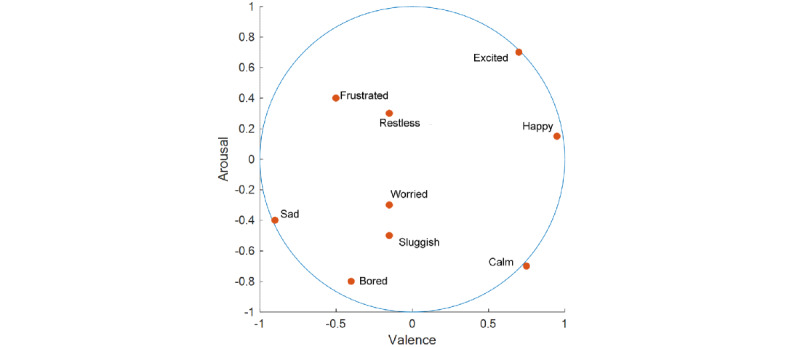
Placement of the 9 emotions considered within the circumplex in the Cartesian coordinate system.

The circumplex model of affect [5,14] was used to categorize emotion in a 2D circular space, containing dimensions for arousal and valence (Figure 1). Valence in the context of emotions means the intrinsic attractiveness or averseness of an event, object, or situation. Likewise, arousal is the state of being physiologically and mentally alert, awake, and attentive. In a recent refinement of the model using regression [16], the circumplex model was quantitatively visualized as a circular space of radius 1 unit within a 2D Cartesian coordinate system, which assigns scores for valence and arousal for each emotion. This model includes a comprehensive list of mood items commonly considered in behavioral sciences. Scores for valence and arousal in the circumplex were obtained from this model for each of the 9 emotion items considered and are shown in Table 1. In Figure 1, these 9 emotions are presented within the circumplex depicting their valence and arousal coordinates. During each daily assessment, composite scores of valence and arousal were created as weighted sums of responses from all the 9 emotion questionnaire items, with the reported emotion scores serving as weights. These 2 composite scores were the outcomes of this study. Specifically, let a participant’s response measured using a Likert-type scale for I feel *happy*, I feel *frustrated*, I feel *sad*, I feel *worried*, I feel *restless*, I feel *excited*, I feel *calm*, I feel *bored*, and I feel *sluggish* be denoted by *l*1, *l*2, *l*3, *l*4, *l*5, *l*6, *l*7, *l*8, and *l*9, respectively. Then, using Table 1, the composite score for valence is 0.89 × *l*1 – 0.6 × *l*2 – 0.81 × *l*3 – 0.07 × *l*4 – 0.04 × *l*5 + 0.7 × *l*6 + 0.78 × *l*7 – 0.35 × *l*8 – 0.22 × *l*9. The corresponding composite score for arousal is 0.17 × *l*1 + 0.4 × *l*2 – 0.4 × *l*3 – 0.32 × *l*4 + 0.29 × *l*5 + 0.71 × *l*6 – 0.68 × *l*7 – 0.78 × *l*8 – 0.5 × *l*9. In Table 2 we have provided the mean of the participant means, SD of the participant means (between participant), and the mean of the participant SDs (within participant) for each emotion. We examined the relationship between the composite valence and arousal scores. We would also like to point out that in purely technical terms, Likert data are provided in an ordinal scale. In our analysis, we are treating all variables with Likert scale as quantitative variables. However, several studies, dating back to the 1930s, consistently show that parametric statistics are robust with respect to potential violations of parametric assumptions in Likert data [50]. Hence, parametric methods can be used without concern for invalid statistical results [50].

**Figure 2:**
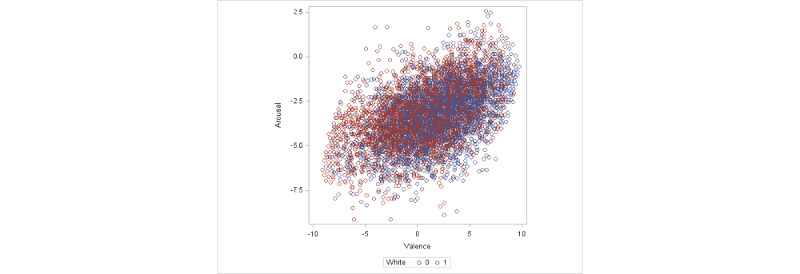
Scatterplot for valence against arousal combining all measurements from all of the participants. White participants and participants of color are color-coded differently in the plot.

**Figure 3:**
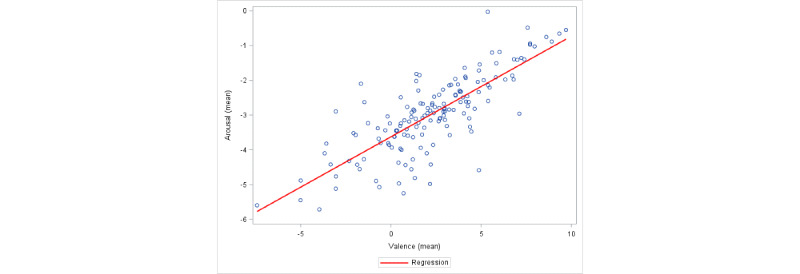
Scatterplot of mean arousal scores against mean valence scores with the fitted regression line.

Our objective was to investigate all 7 forms of the possible relationship described earlier and identify the model that best fits the data. However, the plots of the data at both nomothetic and idiographic levels exhibited a highly dominant positive relationship alleviating the need to consider alternate forms of relationship. In Figure 2, we present a scatterplot of valence against arousal combining all measurements from all participants. White participants and participants of color are color-coded differently in the plot. In Figure 3, we present the scatterplot of arousal against valence scores at the nomothetic level (including the fitted regression line) by calculating the mean valence and arousal scores for each participant over all EMA assessments during the period of participation.

**Figure 4:**
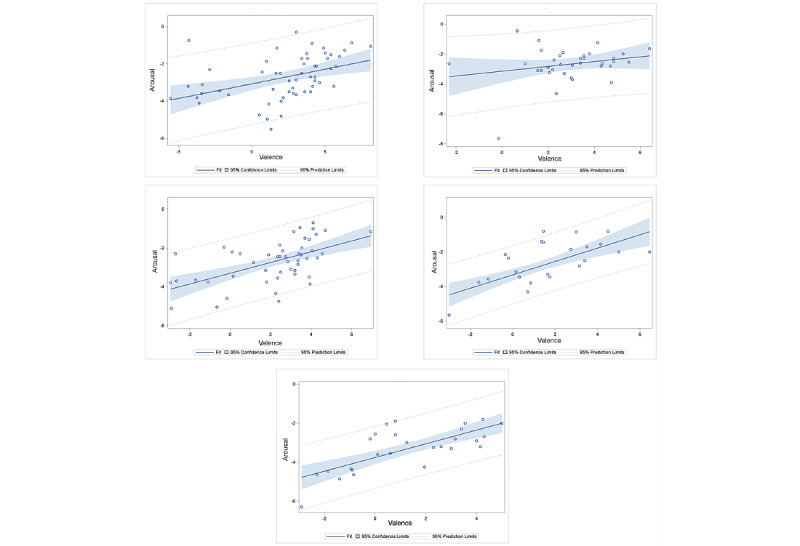
Scatterplot of arousal against valence for 5 random participants in the study. The fitted regression lines along with 95% confidence and prediction limits are also plotted.

Although individual differences were evident, we observed the same linear behavior at an idiographic level. In Figure 4, we present the scatterplot of arousal against valence scores (including the fitted regression line, 95% confidence limits, and 95% prediction limits) separately for 5 representative participants.

**Figure 5:**
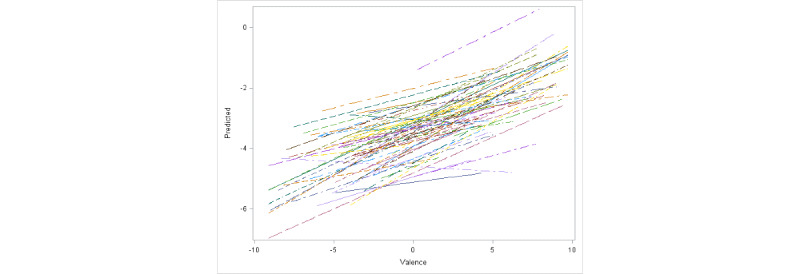
Fitted regression lines for all White participants.

**Figure 6:**
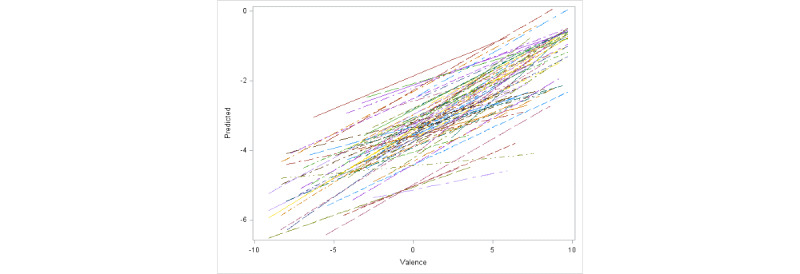
Fitted regression lines for all participants of color.

In a recent work of ours examining the relationship between behavior and affect, we observed a strong difference in affect between White participants and participants of color in this population [28]. To account for potential racial influence on idiographic variations, in Figures 5 and 6, we present the fitted regression lines for all White participants and participants of color, respectively. Each line in the figure corresponds to a single participant. It can easily be observed that although the positive linear relation is evident in both groups, the average slope is much higher for participants of color.

As described before, our primary goal was to study the direct relationship between valence and arousal. Figures 1-6 offer indications of a linear relationship as well as a potential difference in slopes among races.

On the basis of our observations of the plots, we focused on the following linear mixed effects model:

*Arousal_ij_* = (*β*_0_ + *γ*_0_*_i_*) + (*β*_1_ + *γ*_1_*_i_*)*Valence_ij_* + *β*_2_*White_i_* × *Valence_ij_* + *∈_ij_* **(1)**

In equation 1, *i* denotes the *i*-th participant, and *j* denotes the *j*-th measurement from the participant. *β*_0_ and *β*_1_ are the fixed effects for intercept and slope, respectively. The model also includes the participant-specific random intercept and slope denoted by *γ*_0_*_i_* and *γ*_1_*_i_*, respectively. To account for possible differences in slope based on race, a fixed-effect interaction term *White_i_* × *Valence_ij_* with regression coefficient *β*_2_ is included, where *White_i_* is an indicator variable taking the value 1 for a White participant and 0 otherwise.

Finally, *∈_ij_* is the error term assumed to follow a normal distribution with a mean of 0 and unknown, fixed variance. The random effects *γ*_0_*_i_* and *γ*_1_*_i_* are also assumed to follow a normal distribution with mean of 0 and unknown, fixed variances.

To consider any possible fixed effect of race, sex, and an interaction effect of sex with the slope of the linear relationship, we also considered 2 additional models:

*Arousal_ij_* = (*β*_0_ + *γ*_0_*_i_*) + *β*_1_*White_i_* + (*β*_2_ + *γ*_1_*_i_*)*Valence_ij_* + *β*_3_*White_i_* × *Valence_ij_* + *∈_ij_* **(2)**

*Arousal_ij_* = (*β*_0_ + *γ*_0_*_i_*) + *β*_1_*White_i_* + *β*_2_*Sex_i_* + (*β*_3_ + *γ*_1_*_i_*)*Valence_ij_* + *β*_4_*White_i_* × *Valence_ij_* + *β*_5_*Sex_i_* × *Valence_ij_* + *∈_ij_* **(3)**

All 3 models were plausible to capture the linear relationship between valence and arousal. Model 1 is nested within model 2, which in turn is nested within model 3. This allowed us to perform likelihood ratio tests for model optimality. Finally, once the optimal linear mixed effects model was selected, the quality of fit was assessed in 2 different ways. First, a visual assessment was carried out by plotting all the predicted values of arousal based on valence against the observed arousal scores. Second, a simple correlation between the predicted and observed scores was calculated as a descriptive measure of the quality of prediction (adjusted *R*^2^ is not meaningful for mixed effects models). All statistical analyses were conducted using the MIXED procedure in SAS software (SAS Institute Inc), and the restricted maximum likelihood method was used to obtain the estimates that are less biased for finite samples. The maximum likelihood method was used for likelihood ratio tests comparing fixed effects.

**Table 1:** Circumplex scores for the emotions considered.

Emotions	Valence	Arousal
Happy	0.89	0.17
Frustrated	–0.60	0.40
Sad	–0.81	–0.40
Worried	–0.07	–0.32
Restless	–0.04	0.29
Excited	0.70	0.71
Calm	0.78	–0.68
Bored	–0.35	–0.78
Sluggish	–0.22	–0.50

**Table 2:** Descriptive statistics for the 9 emotion outcomes.

Variable	Mean (between-participant SD)	Mean of within-participant SD
Happy	3.54 (0.74)	0.70
Sad	2.55 (0.79)	0.79
Restless	2.59 (0.83)	0.70
Excited	3.09 (0.78)	0.70
Calm	3.42 (0.71)	0.67
Sluggish	2.71 (0.92)	0.74
Frustrated	2.55 (0.79)	0.79
Worried	2.60 (0.85)	0.74
Bored	2.41 (0.80)	0.66

## Results

### Fixed-Effect Solutions From the 3 Mixed Models

The fixed-effect solutions from the 3 mixed models are presented in Tables 3-6. In Table 3 (results from equation 1), we find both the fixed effects of valence and its interaction with race to be highly significant. In Table 4 (results from equation 2), race was included as a fixed effect but offered no evidence of statistical significance. The fixed effects of valence and its interaction with race remained unchanged. In Table 5 (results from equation 3), both race and sexes were included as fixed effects. An interaction term was included to account for the potential difference in slopes among the sexes. As before, only the fixed effects of valence and its interaction with race were statistically significant. These results were consistent with the results from Tables 3 and 4.

**Table 3:** Results for the fixed effects in the mixed effects model corresponding to equation 1.

Effect	Estimate (SE)	*t* test (*df*)	*P* value
Intercept	–3.49 (0.06)	–56.63 (154)	<.001
Valence	0.25 (0.01)	20.28 (149)	<.001
Valence × White	–0.06 (0.02)	–3.23 (153)	.001

**Table 4:** Results for the fixed effects in the mixed effects model corresponding to equation 2.

Effect	Estimate (SE)	*t* test (*df*)	*P* value
Intercept	–3.48 (0.09)	–40.46 (153)	<.001
White	–0.03 (0.12)	–0.23 (149)	.82
Valence	0.25 (0.01)	20.95 (153)	<.001
White × valence	–0.06 (0.02)	–3.19 (153)	.001

**Table 5:** Results for the fixed effects in the mixed effects model corresponding to equation 3.

Effect	Estimate (SE)	*t* test (*df*)	*P* value
Intercept	–3.48 (0.11)	–32.89 (152)	<.001
White	–0.03 (0.12)	–0.22 (153)	.83
Male	–0.004 (0.12)	–0.04 (153)	.97
Valence	0.27 (0.01)	18.47 (148)	<.001
White × valence	–0.06 (0.02)	–3.28 (153)	.001
Male × valence	–0.03 (0.02)	–1.82 (153)	.07

**Table 6:** Random effects estimates of intercept and slope for the first 10 participants.

Effect			Estimate for predicting individual response (SE)		*t* test (*df*)		*P* value
**Participant 1**							
	Intercept	–0.20 (0.27)		–0.77 (153)		.44	
	Valence	0.06 (0.06)		1.03 (153)		.31	
**Participant 2**							
	Intercept	1.64 (0.18)		9.07 (153)		<.001	
	Valence	–0.03 (0.04)		–0.75 (153)		.46	
**Participant 3**							
	Intercept	–1.10 (0.32)		–3.46 (153)		<.001	
	Valence	0.18 (0.05)		3.87 (153)		<.001	
**Participant 4**							
	Intercept	–1.63 (0.10)		–15.98 (153)		<.001	
	Valence	–0.11 (0.03)		–3.49 (153)		<.001	
**Participant 5**							
	Intercept	–0.19 (0.09)		–2.07 (153)		.04	
	Valence	–0.02 (0.04)		–0.57 (153)		.57	
**Participant 6**							
	Intercept	1.28 (0.31)		4.19 (153)		<.001	
	Valence	–0.07 (0.04)		–1.84 (153)		.07	
**Participant 7**							
	Intercept	0.14 (0.12)		1.12 (153)		.26	
	Valence	0.12 (0.03)		3.72 (153)		<.001	
**Participant 8**							
	Intercept	–0.09 (0.15)		–0.59 (153)		.56	
	Valence	–0.06 (0.04)		–1.37 (153)		.17	
**Participant 9**							
	Intercept	0.38 (0.35)		1.08 (153)		.28	
	Valence	0.04 (0.07)		0.66 (153)		.51	
**Participant 10**							
	Intercept	–1.54 (0.11)		–13.86 (153)		<.001	
	Valence	–0.04 (0.06)		–0.68 (153)		.50	

### Best Choice for the Data Set

The results of all 3 models show a dominant linear relation between valence and arousal and a significant difference in the slopes between White participants and participants of color. None of the other effects offered any evidence of statistical significance. Therefore, it is evident that the model corresponding to equation 1, which also happens to be the simplest one, is the best choice for this data set. This was further confirmed by performing likelihood ratio tests for the nested models.

**Figure 7:**
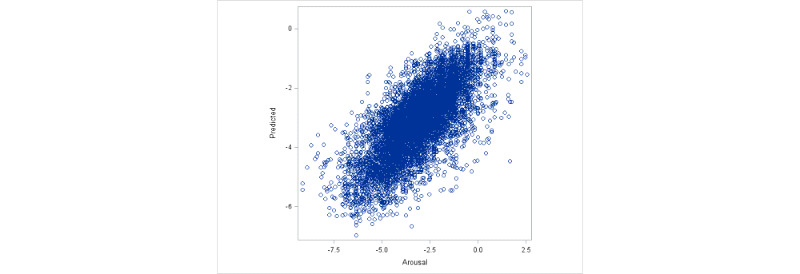
Plot of predicted arousal scores based on the optimal linear mixed effects model against the observed arousal scores.

As the model based on equation 1 was deemed to be the best choice for the data set, we discuss the results based on this model. Both valence and interaction of race with valence are highly significant fixed effects. The fixed effect of valence is estimated to be 0.25, which can be interpreted as the fixed slope of arousal against valence for participants of color (coded as 0 in the model for the variable White). The fixed effect of interaction of valence with race is estimated to be –0.06, which can be interpreted as the fixed change in slope of arousal against valence for White participants compared with participants of color (White participants are coded as 1 in the model for the variable White). Hence, for White participants, the average slope was estimated to be 0.19, which is 0.06 units smaller than the estimated average slope for participants of color. These findings are consistent with Figures 5 and 6, where we clearly see a higher overall slope for participants of color. In Table 6, we present the estimated random intercepts and slopes for the first 10 participants to illustrate individual differences from the fixed effects of intercept and slope. The results indicate strong idiographic differences, with 70% (7/10) of the participants exhibiting significant random effects for either intercept or slope. To make a visual assessment of the quality of prediction from our model, in Figure 7, we present a plot of predicted arousal scores based on the optimal linear mixed effects model against the observed arousal scores. The graph offers strong evidence for the excellent prediction ability of our model. We also calculated a simple correlation between the predicted and observed scores. We obtained a value of 0.81, which indicates a high quality of prediction.

## Discussion

### Principal Findings

#### Overview

We found a significant dominant linear relation between valence and arousal. We also found a significant difference in slopes between White participants and participants of color (predominantly African American participants). There was no evidence that sex played any role in the relationship.

Our findings stand in contrast to the general consensus in several key aspects. In the following sections, we compare our findings with those of the comprehensive study by Kuppens et al [44] and discuss the differences as well as the implications of our findings.

#### Nomothetic Level

For a wide range of data sets, Kuppens et al [44] observed a weak V-shaped relationship between valence and arousal at the nomothetic level but not at the idiographic level. Hence, the authors concluded that any statement about the predicted value of arousal based on the observed value of valence is highly probabilistic. On the basis of this conclusion, it is reasonable to infer that a participant is more likely to be aroused at high levels of pleasure or displeasure. To the contrary, we found a strong linear relationship between valence and arousal at the nomothetic level, and this prediction was possible with high accuracy.

#### Idiographic Level

At the idiographic level, Kuppens et al [44] did not find a consistent relationship between valence and arousal. In fact, their observation was that the relationship can take any form. On the basis of this observation, the authors concluded that no universal relation exists between valence and arousal at the idiographic level. The findings from our study lead us to the opposite conclusion. As observed in Figures 4-6, there is clear evidence of a linear relationship even at the idiographic level, although it is somewhat weaker than the relationship at the nomothetic level. This again suggests the possibility of a universal linear relationship between valence and arousal. Our results also show expected differences in strengths and slopes among participants. This finding may have 2 implications. First, if the linear relation between valence and arousal holds true for a large number of people, it should be possible to identify these individuals by performing psychological tests. Once these individuals are identified, researchers may be able to manipulate arousal simply by manipulating valence or vice versa.

#### Valence as a Function of Arousal

Kuppens et al [44] primarily looked at arousal as a function of valence to study how valence affects arousal. It is also worth investigating how arousal affects valence. In prior studies, it has been difficult to study valence as a function of arousal because arousal was found to be nonmonotone in valence (V-shaped). However, the measurements in prior studies were predominantly made with positive values for valence. Hence, in these studies, for a model that fits valence as a function of arousal, the relationship was deemed linear [35]. As we observed a strong monotone linear relationship when arousal was treated as a function of valence, the same holds true when valence is treated as a function of arousal.

#### Possible Reasons for Discrepancies Between the Findings

As important as our findings may be, it is critical to understand why our findings might conflict with those of Kuppens et al [44]. There are several possible reasons for the discrepant conclusions. First, among the 8 studies previously analyzed, in the first 6 studies, valence and arousal were primarily measured in the presence of visual stimuli, and thus it is conceivable that the observed weak V-shaped relationship is an artifact of visual perception. The last 2 studies did involve affective experience in everyday life; however, in the study involving affective experience in European life, valence and arousal were determined based on participants’ markings on an affect grid (Russell et al [42]). This is different from how we measured affect in our study. Hence, a second possible explanation for the discrepancy is how affect has been measured. In the other study involving affective experience in American life, affect was measured using adjectives as in our study. It is hard to reconcile the discrepant findings, although the composite scores for valence and arousal were calculated differently. Finally, our population of individuals tended to have a range of mental and physical health issues. It is possible that the observed differences are due to sample differences.

### Limitations and Strengths

Our finding of a strong linear relationship between valence and arousal at both the nomothetic and idiographic levels contrasts with the V-shaped relationship found by Kuppens et al [44]. The richness of our EMA data, collected over a relatively long period of time, provides strength to our finding. In fact, the in vivo observations from a reasonably large and diverse sample with a long sampling period is the primary strength of our study. However, the scope of our work is inherently limited because our study population consists of persons residing in PSH who self-reported mental health conditions. High positive arousal with high negative (low) valence as well as high positive valence with high negative (low) arousal were rarely reported in our sample. This may not be the case in other populations. Hence, it is possible that our findings may not be generalizable to other populations. Furthermore, we have observed a significant racial difference in slope for the linear relationship between valence and arousal. However, to our knowledge, there is currently no theoretical construct to explain the difference. Further studies would be necessary to obtain a proper explanation, perhaps by including additional measures of stress or acculturation.

### Conclusions

Our findings provide a window to the fundamental structure of affect. There are 4 broad takeaways from our study. First, we found a strong positive linear relationship between valence and arousal at the nomothetic level. Second, despite obvious individual differences, the relationship broadly held at the idiographic level. Third, the findings open up the possibility that there may be a universal structure of affect. Finally, the study needs to be replicated for different populations to determine whether our findings can be generalized beyond the population studied here.

## Abbreviations

EMA: ecological momentary assessment
IAPS: International Affective Picture System
PSH: permanent supportive housing
